# The American Journal of Obstetrics and Diseases of Women and Children

**Published:** 1871-02

**Authors:** 


The American Journal of Obstetrics and Diseases of Women and Children. Edited by E. Naeggerath, M.D., and B. F. Dawson, M.D. Published by W. A. Townsend & Adams, New York.

We have received from the publishers the first and second volumes of this journal, neatly bound in cloth. Each volume contains four of the quarterly numbers of the *Journal*, and together they embrace the issues for 1868 and 1869. These volumes contain a large amount of valuable matter relating to the diseases peculiar to females, and resulting from pregnancy and parturition. Many of the writers rank among the oldest in our country.

It is a journal well worthy of a place on the table of every practitioner.

